# Linguistic changes in neurodegenerative diseases relate to clinical symptoms

**DOI:** 10.3389/fneur.2024.1373341

**Published:** 2024-03-25

**Authors:** Melisa Gumus, Morgan Koo, Christa M. Studzinski, Aparna Bhan, Jessica Robin, Sandra E. Black

**Affiliations:** ^1^Winterlight Labs, Toronto, ON, Canada; ^2^Department of Psychology, University of Toronto, Toronto, ON, Canada; ^3^Hurvitz Brain Sciences Program, Sunnybrook Research Institute, Toronto, ON, Canada; ^4^School of Public Health Sciences, University of Waterloo, Waterloo, ON, Canada; ^5^Ontario Brain Institute, Toronto, ON, Canada; ^6^Department of Medicine (Neurology), University of Toronto, Toronto, ON, Canada

**Keywords:** speech, linguistic, neurodegenerative diseases, digital health, clinical symptoms

## Abstract

**Background:**

The detection and characterization of speech changes may help in the identification and monitoring of neurodegenerative diseases. However, there is limited research validating the relationship between speech changes and clinical symptoms across a wide range of neurodegenerative diseases.

**Method:**

We analyzed speech recordings from 109 patients who were diagnosed with various neurodegenerative diseases, including Alzheimer’s disease, Frontotemporal Dementia, and Vascular Cognitive Impairment, in a cognitive neurology memory clinic. Speech recordings of an open-ended picture description task were processed using the Winterlight speech analysis platform which generates >500 speech features, including the acoustics of speech and linguistic properties of spoken language. We investigated the relationship between the speech features and clinical assessments including the Mini Mental State Examination (MMSE), Mattis Dementia Rating Scale (DRS), Western Aphasia Battery (WAB), and Boston Naming Task (BNT) in a heterogeneous patient population.

**Result:**

Linguistic features including lexical and syntactic features were significantly correlated with clinical assessments in patients, across diagnoses. Lower MMSE and DRS scores were associated with the use of shorter words and fewer prepositional phrases. Increased impairment on WAB and BNT was correlated with the use of fewer nouns but more pronouns. Patients also differed from healthy adults as their speech duration was significantly shorter with more pauses.

**Conclusion:**

Linguistic changes such as the use of simpler vocabularies and syntax were detectable in patients with different neurodegenerative diseases and correlated with cognitive decline. Speech has the potential to be a sensitive measure for detecting cognitive impairments across various neurodegenerative diseases.

## 1 Introduction

Speech and language impairments are a prominent symptom in many neurodegenerative diseases including Alzheimer’s Disease (AD), Frontotemporal Dementia (FTD) and Vascular Dementia (VD) (1–5). These changes emerge along with the primary set of symptoms such as memory deficits and unusual behaviors associated with each neurodegenerative disease. There have been efforts in differentiating various dementias from healthy older populations based on speech and language assessments (1, 4, 6–10). However, many patients with dementia diagnoses exhibit comorbid conditions. The manifestation of symptoms could differ dramatically from one to another patient despite having received the same diagnosis, making characterization of disease incredibly complicated. Nevertheless, beyond the diagnosis of the patient, each neurodegenerative disease presents multiple symptoms that progress differently in each individual. Thus, understanding how speech and language changes manifest themselves in neurodegenerative diseases and how they relate to clinical symptoms can help add to the clinical picture and improve characterization of neurodegenerative disease. Here, we investigate this question by leveraging a rich, heterogeneous dataset that includes a wide range of neurodegenerative disease diagnoses.

Speech and language changes are an integral part of symptom progression in neurodegenerative diseases such as in AD. Although symptoms of typical AD concern deficits in episodic memory, executive function or reasoning, patients might also experience language impairments, specifically, in semantic abilities, verbal fluency or language comprehension (1, 8, 11, 12). These language deficits manifest themselves as impairments in verbal naming, speech pauses and word finding ability (13–15). Atypical AD, specifically the logopenic variant of primary progressive aphasia (PPA), is associated with impaired lexical access, naming difficulty, dysfluencies (16–19). Recent efforts identified a set of language changes, specifically in lexical retrieval and fluency, that differed between the logopenic variant of PPA and the typical AD although these were not related to clinical outcomes (20). Nevertheless, typical AD and the logopenic variant of PPA share certain language deficits such as production of more adverbs, fewer prepositions and nouns (20). In fact, many neurodegenerative diseases show overlapping speech and language changes, emphasizing the importance of understanding their relation to cognitive decline.

Frontotemporal Dementia (FTD) encompasses distinct cognitive, social and behavioral symptoms that differ from AD, yet speech changes have also been reported across subtypes. Clinically, FTD could present itself as the behavioral variant or PPA, which could be further subdivided into fluent (semantic) and non-fluent variants (21). Patients with the non-fluent variant exhibit deficits in prosody, articulation and local coherence of speech (22–24). Thus, the symptoms include effortful speech, morphological or syntactic deficits, and word finding difficulty that result in simplified language (1). Semantic dementia (fluent variant), on the other hand, is characterized by decline of conceptual knowledge (i.e., semantic memory), primarily involving word comprehension (25). These patients lose the meaning of the words, mostly, nouns, but speech fluency and phonology stays relatively intact (26). As a result, patients with semantic dementia tend to produce fewer but more familiar nouns, and more adverbs than healthy adults (9). Lastly, patients with the logopenic variant of PPA, who tend to show AD pathology at autopsy, exhibit word finding difficulty, phonemic errors, and increased number of pronouns (16, 19, 22). In contrast to fluent and non-fluent variants of FTD, semantic memory, grammar, syntax and production of speech remain relatively unaffected in these patients (16, 19, 22). Lastly, speech and language change in naming, single word or discourse comprehension, and prosody in the behavioral variant of FTD although, historically, this variant was not considered to present such symptoms (27–30). The heterogeneity across different variants of FTD complicates the characterization of distinct speech and language differences for each disease.

Overlapping speech and language deficits across neurodegenerative diseases blur the diagnostic boundaries. Flow of speech as known as fluency could depend on the stage of the disease, in other words, the degree of cognitive decline (31). Fluency changes are common in AD although it remains unclear whether these reflect deficits in semantic knowledge, accessing or retrieving information while producing speech (32, 33). Early onset of AD often exhibits similar speech and language deficits as mild cognitive impairment, primarily in lexico-semantic domain such as access to semantic knowledge or lexical decisions (12, 34, 35). Language changes including difficulty with name generation and single word comprehension in AD are also common in semantic dementia (i.e., fluent FTD). Although single word comprehension is spared in logopenic progressive aphasia, speech rate tends to be slow due to slow word retrieval and frequency pauses to find the right words (17). In fact, logopenic variant aphasia can exhibit AD pathology or progress into dementia caused by AD in later stages (36, 37). Thus, similar speech and language changes may occur across different neurodegenerative diseases depending on the disease stage, social factors, and co-morbid condition.

Heterogeneity in and across neurogenerative diseases necessitates the development of digital speech and language biomarkers that can capture the cognitive decline and clinical symptoms, extending beyond the diagnostic categories. Literature reveals overlapping clinical symptoms and speech changes across neurogenerative diseases. For example, semantic dementia or progressive non-fluent aphasia could present different clinical symptoms, but both patient groups use significantly shorter words than healthy controls (9). However, the word length does not differ between the patients group (9). Similarly, both AD and semantic dementia (i.e., fluent FTD) may exhibit difficulty with name generation or word comprehension (12, 25, 38). Distinct diagnoses may share similar speech and language symptoms although the underlying pathology or affected cognitive domains may differ. Thus, understanding how speech and language changes are linked to cognitive impairment and clinical symptoms beyond the diagnosis labels is as crucial as distinguishing patients from healthy controls. As speech and language changes occur across a variety of neurodegenerative diseases, speech assessments might not be as powerful for differentiating diagnoses, but relate more to cognition, function, and clinical outcomes across diseases. This would mean that speech might be more useful for a cross-diagnosis cognitive assessment, rather than a sole diagnostic tool.

Speech and language features have the potential to be used as a screening or symptom-monitoring tool for neurodegenerative diseases. There is an added benefit of the feasibility of collecting speech samples remotely and with high frequency. We recently demonstrated the importance of high frequency speech assessments in understanding the individual symptom fluctuations relating to depression and cognitive impairment (30, 39, 40). In neurodegenerative diseases, there are no standardized speech and language assessments to be utilized. Thus, short, automated speech assessments which can be administered remotely and with high frequency can be powerful tools in understanding disease progressions. Machine learning and natural language processing models could be used to classify patients with dementia using speech features or differentiate them from healthy controls. Yet, the challenge is to interpret the speech and language changes in terms of the clinical symptoms. Here, our aim is to determine how speech and language changes relate to clinical outcomes in neurodegenerative diseases, beyond the diagnostic categories. Thus, we leveraged a rich, heterogenous patient sample with many neurodegenerative diseases and comorbid diagnoses and investigated the link between clinical outcomes and hundreds of speech features derived through natural language processing. Understanding how potential speech and language biomarkers relate to clinical symptoms will enable early detection or monitoring of cognitive decline at the individual level, as a way of addressing heterogeneity in neurodegenerative diseases.

## 2 Methods

### 2.1 Participants

The part of this study related to the patient cohort was approved by the Sunnybrook Research Ethics Board. Patients were recruited from the memory clinics at Sunnybrook Health Sciences Center in Toronto, Ontario, Canada. An informed consent discussion was conducted with all research candidates; all of these candidates agreed to participate, provided a written consent, and were successfully enrolled in the study. For our additional analyses, we also included a healthy older adult sample to compare timing related speech features to patients. The healthy control arm of this study was approved by the Advarra Research Ethics Board. Healthy controls were recruited from the community. Informed consent was collected from all participants. To be eligible for the study, participants needed to be between the ages of 50–95 and fluent English speakers (i.e., either English as their first language or they can speak with conversational proficiency). The exclusion criteria included the following: residing outside of Canada or the United States and having diagnosis of dementia, memory impairment, recent concussion or traumatic brain injury, or uncorrected hearing or visual impairment.

### 2.2 Clinical assessments

Patients completed a series of cognitive assessments including the Mini Mental State Examination (MMSE) (41), Dementia Rating Scale (DRS) (42), Western Aphasia Battery (WAB) (43), and Boston Naming Test (BNT) (44). MMSE and DRS have been administered to assess cognitive impairment in patients while WAB and BNT have been used to assess speech and language related changes. WAB is helpful in characterizing many different aspects of speech and language including fluency, comprehension, naming, reading and writing. BNT is mostly used to assess retrieval of lexical information while naming an object. WAB and BNT were used to validate our speech features and determine how they relate to specific speech changes. On the other hand, MMSE and DRS were used to assess the cognitive decline and its relationship to speech and language changes detected with our extensive features.

### 2.3 Acoustic and linguistic speech features

Patients’ speech recordings were collected in the clinic while they were performing a picture description task as part of WAB. The picture that participants described was a line drawing of a picnic scene. Healthy controls completed the picture description task as well, but using an app-based interface and a picture of a family in the kitchen scene. The recordings from healthy controls were collected with the Winterlight Speech App. Because of the significant differences between the 2 stimuli, we only analyzed the timing related speech features for the comparison between the patients and healthy controls since linguistic differences of spoken language could relate to the different picture content.

The patients’ speech recordings were transferred to the Ontario Brain Institute’s “Brain-CODE” informatics platform^1^ for processing and analysis. “Brain-CODE” was designed to support the collection, integration, sharing, and analysis of patient-level data, while abiding by ethical principles and government legislation (45).

First, a trained transcriptionist transcribed and labeled the speech samples, ensuring the quality of audio content and flagged any recordings with significant issues for removal, such as no audible speech or poor audio quality. Although speech recordings were transcribed for quality purposes, the analyses are based on the recorded spoken speech. To make the distinction between speech and language, here acoustics refer to the auditory features of speech while the linguistics relates to the spoken language. Speech samples were then passed through the Winterlight Lab processing pipeline, which relies on python-based language processing libraries and proprietary custom code. Open source packages include SpaCy for parts-of-speech tagging and morphological features (46), the Stanford NLP parser for syntactic features (47), Praat and Parselmouth for acoustic features (48, 49), and GloVe and FastText models for semantic features (50, 51). The pipeline also uses custom code to compute additional features based on the transcript and audio file, using lexical norms from previous publications (52–55) or previously published models and features (56).

The Winterlight Lab processing pipeline enables us to extract 707 speech features from the audio files and transcripts. These features reflect various aspects of speech: acoustics (e.g., properties of the sound wave, speech rate, number of pauses), lexical content (e.g., rates and types of words used, and their characteristics such as frequency and imageability, which reflect how commonly words are used and how easy they are to picture, respectively), semantics (relating to the meaning of the words, e.g., semantic relatedness of subsequent utterances, semantic relatedness of utterances to the items in the picture) and syntactic (relating to the grammar of the sentences, e.g., syntactic complexity, use of different syntactic constructions) properties of the recordings. While it is not possible to review each feature in detail, we provide more detailed definitions for some of the features of interest in this paper. For instance, average word length and noun or pronoun count fall under the lexical category. Average word length represents the mean number of letters in each word used to describe the picture. Noun and pronoun count are calculated based on the number of nouns or pronouns in a transcript divided by the number of words, respectively. Lexical features also include noun familiarity and frequency, which represent the mean familiarity or frequency of the nouns in the transcript, based on familiarity or frequency norms, respectively (52, 54). In other words, familiarity is a subjective rating of how common the word is, and frequency is how often it appears in a standard corpus of speech. Prepositional phrase count, as an example of syntactic features, is the number of times the phrase occurs in a transcript. Timing related features include mean speech or pause duration. Speech duration is the total number of seconds that participants take to describe the picture while pause duration is calculated by dividing the duration of unvoiced segments of speech by the total number of unvoiced segments. An overview of the feature categories, definitions, numbers, and examples is provided in Table 1.

**Table 1:** Speech feature overview, definitions, numbers, and examples.

Speech feature category	Definition	Number of features	Examples
Acoustic	Variables describing the acoustic properties of the sound wave	209	Fundamental frequency; Mel-frequency Cepstral Coefficients (MFCCs); zero crossing rate
Timing	Variables relating to the rate of speech and total speech output	11	Speech rate (words/min); articulation rate (syllables/s); number of pauses; pause duration; total duration of speech
Parts of speech	Variables enumerating the rate of usage of different parts of speech	178	Use of nouns, pronouns, verbs, adjectives, etc.
Lexical	Variables describing the characteristics of words used	104	Frequency, familiarity, imageability of words; measures of vocabulary diversity such as type-token ratio
Syntactic	Variables enumerating the rate of usage of different syntactic structures and measures of syntactic complexity	163	Number of clauses per sentence; use of noun phrases, verb phrases, subordinate phrases, etc.
Discourse	Variables using cosine distance and graph theoretical measures to calculate the organization and repetition of utterances	18	Average cosine distance between utterances; graph density, number of nodes and diameter
Coherence	Variables using word vector models to calculate the semantic similarity between utterances	15	Average, minimum and maximum cosine distances between subsequent utterances in word vector space
Sentiment	Variables describing the sentiment of the words used	9	Valence, arousal and dominance scores for all words and word types

### 2.4 Statistical analyses

All analyses were completed on R statistical software, version 4.1.2 (57). We eliminated the speech features that had empty values for at least 20% of participants. 80% of the features eliminated fell under morphological or syntactic speech categories. Specifically, a total of 146 morphological and 94 syntactic speech features had empty values, because of a combination of adverb and prepositional phrases that did not occur in all transcripts, and thus were eliminated from the analyses. Some of the remaining 62 speech features eliminated were related to tags for specific words, for instance, the words with a hyphen (e.g., t-shirt) in them. Most of the remaining speech categories in Table 1 were not affected from this cleaning process, yielding 405 features in total.

We fit separate linear mixed effects models to each speech feature to investigate their unique relationship with the clinical assessments, with covariates of age, sex, and years of education. These analyses were done separately for each of the cognitive assessments; MMSE, DRS, BNT and WAB. Statistical significance was set to an alpha level equal to 0.0001 taking multiple comparisons into consideration through Bonferroni correction (0.05/405 features). Using a series of analysis of variances (ANOVAs), we have compared different diagnoses in terms of the speech feature that we identified in the above analyses. However, these analyses did not yield significant results due to small sample size in each diagnosis.

Lastly, we included a healthy control dataset to be able to compare the speech changes in patients. We only included the timing related features for this comparison. As mentioned above, acoustic and linguistic features might not be appropriate for the comparison because the healthy older adults described a different picture, and recordings were obtained using different devices (digital vs. analog) and recording conditions. We used a Principal Component Analysis (PCA) to reduce the dimensionality of the feature set and distinguish the patients from healthy controls based on the resulting composite variables. This was used as an exploratory cluster analysis. We focused on the first few principal components, that, when combined, were able to explain at least 80% variance. Although there is no straight forward way to determine the number of principal components to retain, the first few components are expected to explain at least 75% of variance or even less in some situations (58–60).

## 3 Results

### 3.1 Demographics

This study included 109 patients (52 females, 57 males) with an age range of 51–91 (M = 72.63 ± 8.61). Patient diagnoses included AD, FTD and Vascular dementia. The largest subset of patients was those diagnosed with AD and familial AD, consisting of 34 patients (Supplementary Table S1). All patients completed MMSE, DRS, WAB and BNT as well as the picture description task as part of WAB (Table 2). We also included 74 healthy controls (39 females) with mean age 61.31 ± 7.29. Healthy controls only completed the picture description task, but no additional clinical assessments reported here.

**Table 2:** Patient demographics.

Demographic variable	Patients (*N* or Mean ± SD)
Sample size	109
Age	72.63 ± 8.61
Sex	52 Females
Education	14.80 ± 3.93
MMSE	22.60 ± 4.85
DRS total	113.75 ± 15.53
WAB total	83.84 ± 12.22
BNT total	15.31 ± 6.42

### 3.2 MMSE and DRS relate to linguistic features

**Figure 1:**
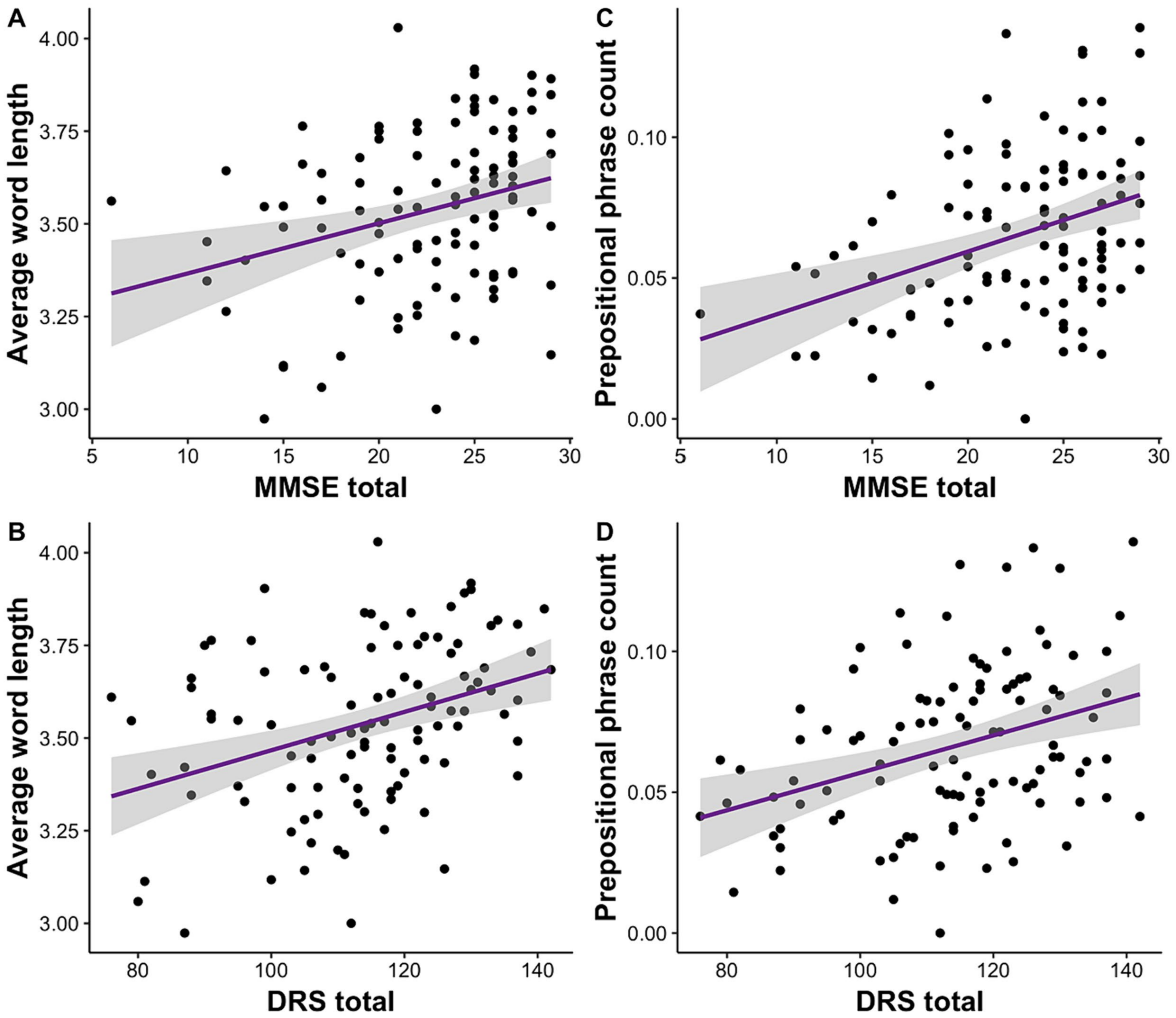
Clinical impairment measured with MMSE and DRS is correlated with average word length and prepositional phrase usage. **(A)** Lower MMSE and **(B)** DRS scores are associated with shorter word length. **(C)** Lower MMSE and **(D)** DRS are also correlated with use of fewer prepositional phrases.

We investigated the relationship of clinical scores on MMSE and DRS to acoustic and linguistic speech features. We conducted separate linear mixed effects models for each speech feature and repeated the analyses for MMSE and DRS separately. We observed that of all the features, average word length was significantly associated with MMSE, *R*^2^ = 0.14, *F* (4, 103) = 5.46, *β* = 0.01, *p* = 0.001, 95% CI [0.005, 0.02], and DRS total scores, *R*^2^ = 0.20, *F* (4, 100) = 7.35, *β* = 0.005, *p* = 5.82E-05, 95% CI [0.003, 0.008] (Figures 1A,B). Similarly, prepositional phrase count was also significantly correlated with MMSE, *R*^2^ = 0.14, *F* (4, 103) = 5.53, *β* = 0.002, *p* = 0.0001, 95% CI [0.001, 0.003], and DRS total scores, *R*^2^ = 0.13, *F* (4, 100) = 5.03, *β* = 0.0007, *p* = 0.0002, 95% CI [0.0003, 0.001] (Figures 1C,D). All of these results remained significant following Bonferroni correction.

Additional results included that the use of familiar nouns was inversely associated with MMSE, *R*^2^ = 0.14, *F* (4, 103) = 5.20, *β* = −1.60, *p* = 0.0001, 95% CI [−2.40, −0.80], and DRS total scores, *R*^2^ = 0.14, *F* (4, 100) = 5.42, *β* = −0.51, *p* = 0.0001, 95% CI [−0.76, −0.26]. Similarly, using frequent nouns were negatively correlated with MMSE, *R*^2^ = 0.14, F(4, 103) = 5.29, *β* = −0.02, *p* = 0.00005, 95% CI [−0.03, −0.01], and DRS total scores, *R*^2^ = 0.12, *F* (4, 100) = 4.21, *β* = −0.006, *p* = 0.0005, 95% CI [−0.01, −0.003].

### 3.3 WAB and BNT relate to linguistic features

**Figure 2:**
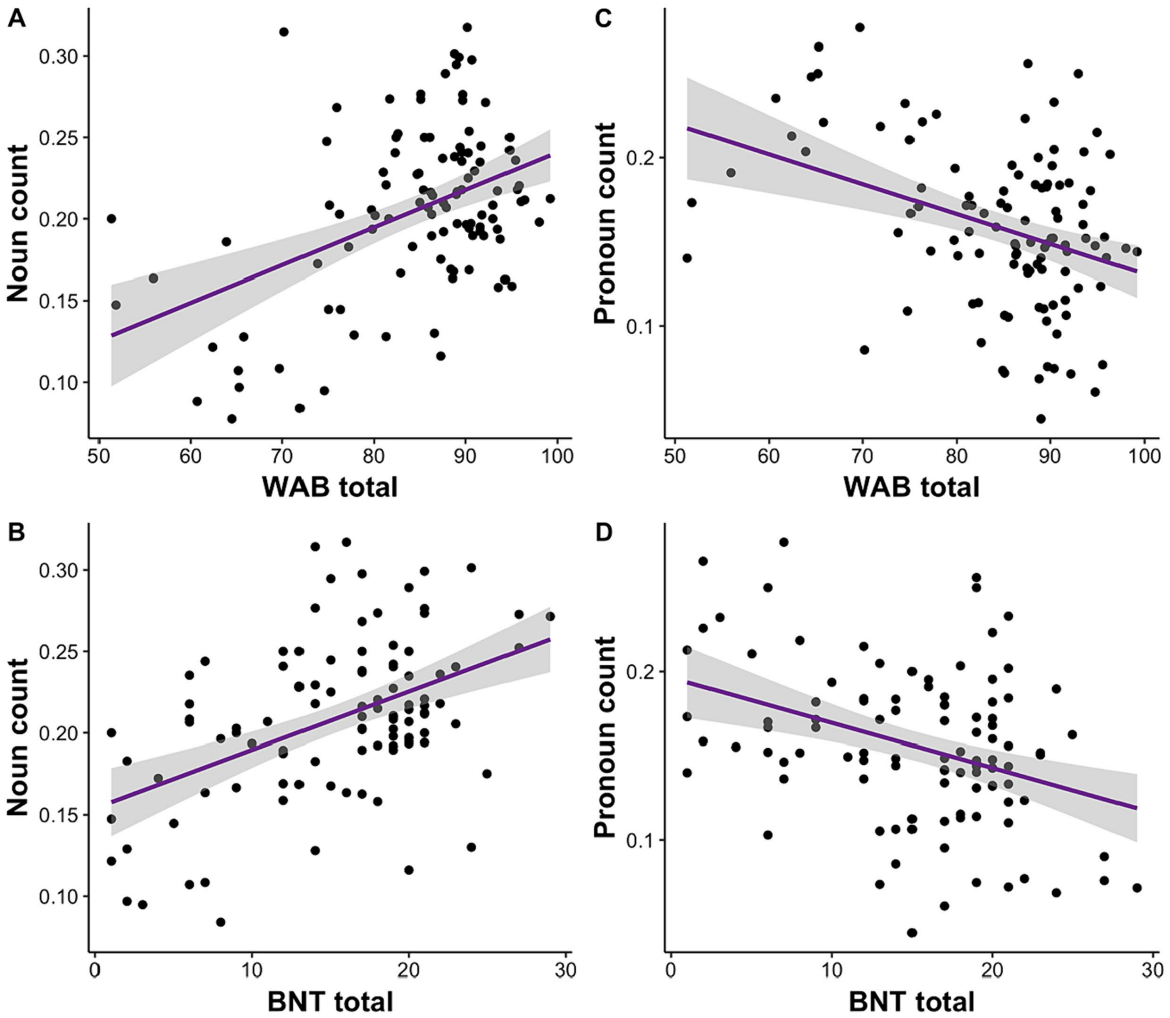
Impairment in language (WAB) and semantic processing (BNT) is associated with the amount of noun and pronoun usage. **(A)** Lower WAB total and **(B)** BNT scores are correlated with reduced noun usage. Conversely, **(C)** lower WAB total and **(D)** BNT scores relate to higher pronoun usage.

We investigated the relationship of language deficits reported on WAB and BNT to acoustic and linguistic speech features. We conducted separate linear mixed effects models for each speech feature and repeated the analyses for WAB and BNT separately. We observed that noun count was significantly associated with WAB, *R*^2^ = 0.16, *F* (4, 104) = 6.13, *β* = 0.001, *p* = 0.0002, 95% CI [0.0007, 0.002], and BNT total scores, *R*^2^ = 0.24, *F* (4, 98) = 8.95, *β* = 0.005, *p* = 1.25E-06, 95% CI [0.002, 0.005] (Figures 2A,B). On the contrary, pronoun count was negatively correlated with WAB, *R*^2^ = 0.09, *F* (4, 104) = 3.63, *β* = −0.001, *p* = 0.002, 95% CI [−0.002, −0.0005], and BNT total scores, *R*^2^ = 0.11, *F* (4, 98) = 4.25, *β* = −0.003, *p* = 0.0003, 95% CI [−0.004, −0.001] (Figures 2C,D).

### 3.4 Timing features distinguish patients from healthy older adults

**Figure 3:**
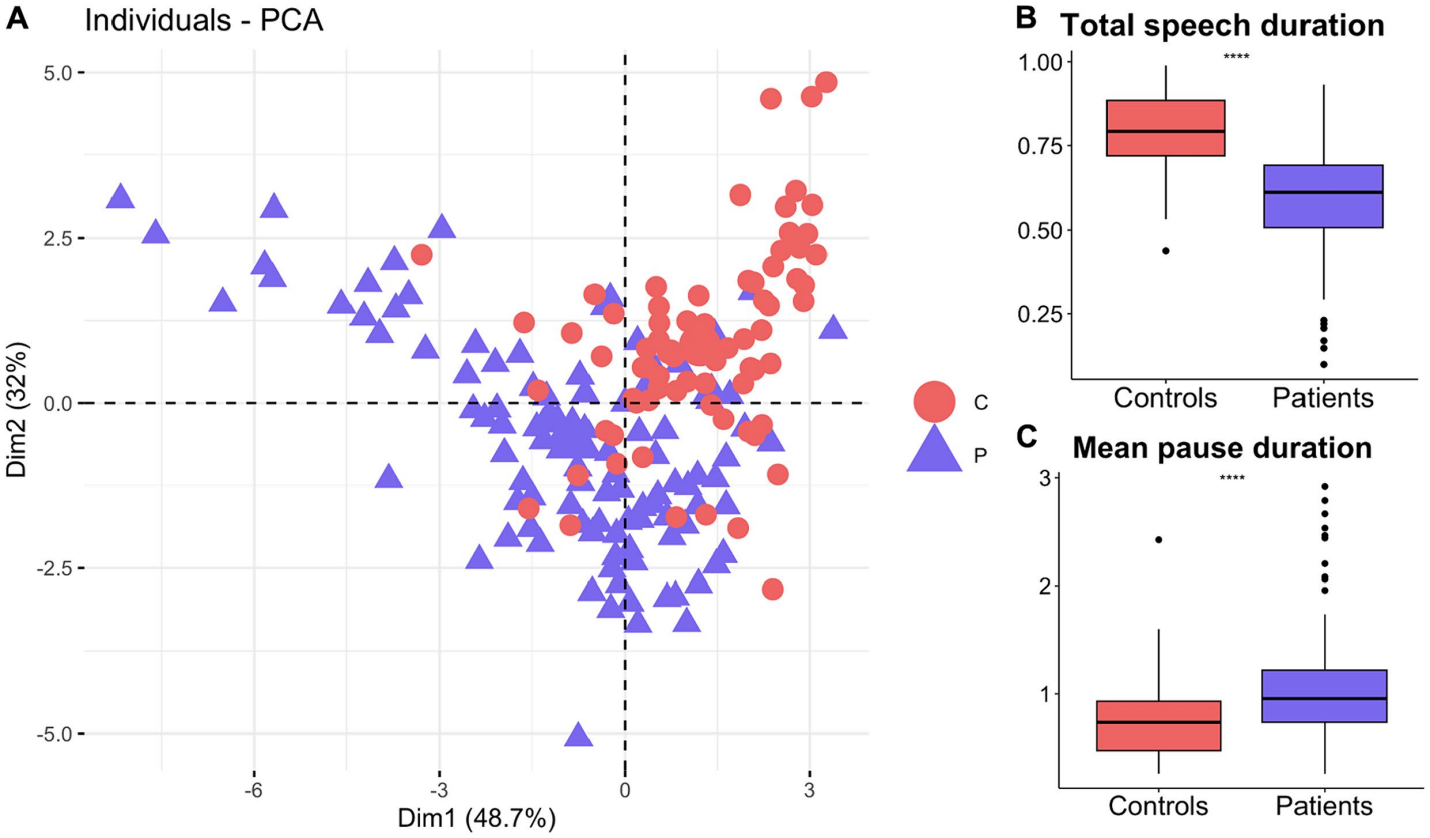
Timing related speech features distinguish patients with neurodegenerative diseases from healthy older adults. **(A)** First 2 principal components based on timing features distinguish patients from healthy controls. Patients produced significantly **(B)** shorter speech durations but **(C)** longer pauses than healthy controls.

Principal component analysis based on timing related features, including duration of speech and pauses, differentiated patients from healthy older adults (Figure 3A). The first two principal components explained more than 80% of the variance within the data (Figure 3A). Mean pause and total speech duration had two of the highest loadings and were used to show the differences between the patients and healthy controls. According to the student t-test, patients (M = 0.59, SD = 0.16) produced significantly shorter speech than the healthy controls (M = 0.79, SD = 0.12), *t* (180.41) = −9.63, *p* = 2.2E-16 (Figure 3B). On the other hand, patients (M = 0.95, SD = 0.49) paused significantly more than the healthy controls (M = 0.75, SD = 0.37), *t* (182.05) = −3.36, *p* = 0.001 (Figure 3C).

## 4 Discussion

This study reports that clinical symptoms in a wide range of neurodegenerative diseases are linked to digital linguistic speech features. Many of the speech and language changes that distinguish patients from the healthy older adults might be overlapping between different neurodegenerative diseases (1). We leveraged a rich and heterogeneous patient sample with many diagnostic labels to investigate how digital speech measures relate to cognitive impairment as well as the linguistic deficits measured with traditional assessments. We show that patients with neurodegenerative diseases tend to use simpler vocabulary and syntax; shorter words and fewer prepositional phrases, reflecting cognitive impairment. They utilize fewer nouns and mostly those that are familiar or frequently used in everyday life. In fact, there appears to be a tradeoff between reduced nouns and increased pronouns, which could result in less specific language. While a healthy older adult might describe a picnic scene as “A lady is sitting on the grass and pouring a beverage next to a gentleman by the lake,” a patient with dementia might only say “She is sitting and pouring something. There is a man.” Building on our previous work looking at longitudinal linguistic changes in AD and FTD (30, 40), the link between linguistic changes and cognition across diagnoses highlights the importance of these language properties beyond the neurodegenerative disease categories.

Our results revealed that the cognitive impairment measured with MMSE and DRS was correlated with use of shorter words on average. Shorter word length is reported in many neurodegenerative diseases including AD, semantic dementia, progressive non-fluent aphasia, behavioral variant of FTD (9, 40, 61, 62). Those with semantic dementia and progressive nonfluent aphasia, for example, may differ from healthy controls in terms of word length, yet the 2 patient groups were not previously found to differ from each other (9). Thus, it is necessary to understand how word length is linked to cognitive impairment in addition to distinguishing patients from healthy controls. Our analyses on a heterogenous patient sample revealed a link between word length and cognitive impairment, suggesting the relationship is beyond the diagnostic labels. This is in line with recent findings that shorter word usage in typical AD and the logopenic variant of PPA is correlated with MMSE and BNT (20). Patients with different neurodegenerative diseases may exhibit common linguistic changes, possibly emerging from different underlying deficits. Change in word length might arise from top-down lexical and semantic processing. Unavailability of long, sophisticated words might also result in word finding difficulty, which is commonly reported across diseases (1, 19, 22).

Since the earlier work, naming difficulty has been linked to word frequency and familiarity in dementia (63, 64). Noun frequency and familiarity are two related linguistic features reflecting vocabulary complexity, and have been previously shown to be impacted in semantic dementia and, more generally, in FTD (30, 65). These features could even distinguish semantic dementia from healthy controls and patients with progressive non-fluent aphasia (9). Besides FTD, we report the involvement of noun frequency and familiarity across many neurodegenerative diseases. These linguistic features are also closely linked to cognitive impairment on MMSE and DRS. Similarly, noun frequency in conversation was recently reported to be implicated in AD (66). However, the frequency was reported to decrease with age, suggesting increased use of more rare or complex words with increased age (66). It was speculated that the frequency measure could vary with education (66, 67). Thus, the varying sensitivity of linguistic features also adds to the heterogeneity in dementia. This emphasizes the importance of understanding the link between linguistic changes and cognitive impairment, not specifically at the diagnostic level, but at the individual level.

Syntactic properties might be less sensitive to dementia than lexical features such as word length or noun frequency (68). We report that patients use fewer prepositional phrases – a syntactic feature - as cognitive impairment increases. Overall, prepositional phrases are relatively less studied, but they could be contributing to fragmented sentences in neurodegenerative diseases (68, 69) or a reduced ability to form connections between concepts. Typical AD and the logopenic variant of PPA produce fewer prepositions than healthy controls (20). Similarly, patients with FTD use fewer and fewer prepositional phrases over time (30). Prepositional phrases in AD were associated with performance on BNT, relating to the difficulty in efficiently retrieving semantic information (20). Interestingly, while prepositional phrase count was related to MMSE and DRS scores in our study, we identified distinct linguistic features relating to traditional language assessments such as BNT and WAB.

With increased deficits on WAB and BNT, we observed decreased use of nouns but increased number of pronouns in patients with neurodegenerative diseases. Linguistic impairments measured with WAB were associated with the severity of dementia across diagnoses such as behavioral variant of FTD, primary progressive aphasia and AD (70). Patients with typical AD or the logopenic variant of PPA produce fewer nouns (20), and the use of nouns in AD and FTD decreases over time (30, 40). Semantic dementia also presents with decreased noun use, specifically in connected speech (9). Supporting these findings, we showed that decreased noun use along with increased pronouns across neurodegenerative diseases was associated with language deficits measured with WAB and BNT. This might indicate semantic deficits (65), which might extend to other neurodegenerative diseases beyond semantic dementia as our results suggest. Indeed, BNT relies heavily on semantic memory (71) and is thus more correlated with category fluency such as knowledge of words rather than the rhyming of words or verbal fluency (32, 33). This could be why BNT performance is linked to nouns and pronouns across diseases in the current study as these features are capturing more of the semantic processing.

Characteristics of speech production such as rate and timing are also affected in neurodegenerative diseases. Our results show that patients produced significantly shorter speech and more pauses in speech than the healthy older adults. This might indirectly be an indication of lexico-semantic deficits in these patients and relate to our findings discussed above. Patients with dementia produced shorter speech recordings than healthy controls (20, 72). Similarly, those with logopenic variants of PPA or AD pause more or longer (14, 17, 20, 73). Increased pauses are observable in MCI and early AD as well (74). Between-utterance pauses were also shown to differ between AD and MCI, and was related to episodic memory performance, suggesting its importance in early detection of symptoms (14). Supporting this, we provided evidence that timing features distinguished healthy controls from the heterogenous patient sample that included more than 10 different diagnoses and various comorbid conditions. Yet, we did not observe any associations between number of pauses and MMSE or DRS. This might suggest that timing of speech could be involved in specific cognitive domains rather than overall cognitive decline. This emphasizes the importance of understanding how each speech and language change is linked to a particular clinical outcome beyond the clinical diagnoses, which could be the key in unpacking the heterogeneity and comorbidity seen in neurodegenerative diseases.

We were not able to distinguish the linguistic differences between the diagnostic groups due to small sample size in each group. Although many neurodegenerative diseases show a great overlap in terms of speech changes, future research should investigate the relationship between linguistic speech changes and various clinical outcomes in each disease population separately. These identified language features here can be the first step in understanding how linguistic changes manifest themselves as cognitive impairment progresses in each disease. In addition to linguistic changes, acoustic aspects of speech have recently been part of the efforts in capturing speech changes in neurodegenerative diseases. We generated a wide range of acoustic features including but not limited to the power spectrum of speech signals, speech intensity, and jitter. Yet, acoustic features were not significantly correlated with clinical scores. The speech recordings were collected during clinical interviews on relatively older devices and on a platform other than the Winterlight App. Future research should investigate the acoustic features in high quality speech samples collected with more up-to-date devices.

This study identified several linguistic features that are linked to cognitive impairment in neurodegenerative diseases. It suggested that certain linguistic features may relate to cognition more generally, others to language abilities while timing related features were best suited for broadly distinguishing patients and healthy controls. In particular, it could be that word length and prepositions relate more to cognitive abilities in general, nouns and pronouns relate more specifically to language abilities and speech duration/pausing distinguishes controls from patients. Although these results alone are not enough to make a strong argument, future research should explore this idea that perhaps we could use one assessment to derive different features to inform on different aspects of cognition/language. Gold standard assessments for cognitive impairment can be laborious to conduct as they require expertise and time. Speech assessments, on the other hand, are automated, fast and could be administered in addition to the existing clinical assessments. Nevertheless, many neurodegenerative diseases including various variants of AD and FTD present overlapping speech and language changes (1). There have been efforts in understanding disease specific changes, yet individual differences make this investigation complicated. Each patients’ medical history, severity level, lifestyle, and cognitive resource might be contributing to their phenotype in different ways. A recent review suggests that neurodegenerative diseases should be considered as a multi-faceted condition that involves biology, psychology and social levels to explain the resulting digital phenotypes (75). We can get one step closer to a more objective understanding of cognitive decline in neurodegenerative diseases through development of digital linguistic biomarkers that link to specific cognitive deficits.

## Data availability statement

The raw data supporting the conclusions of this article will be made available by the authors, without undue reservation.

## Ethics statement

The studies involving humans were approved by 1. Sunnybrook Research Ethics Board or 2. Advarra Research Ethics Board. The studies were conducted in accordance with the local legislation and institutional requirements. The participants provided their written informed consent to participate in this study.

## Author contributions

MG: Formal analysis, Visualization, Writing – original draft, Writing – review & editing. MK: Data curation, Writing – review & editing. CMS: Resources, Writing – review & editing. AB: Project administration, Writing – review & editing. JR: Supervision, Writing – review & editing. SEB: Conceptualization, Supervision, Writing – review & editing.

## Footnotes

^1^ www.braincode.ca
