# Supplementary material for: Linguistic changes in neurodegenerative diseases relate to clinical symptoms
Source: Front Neurol. 2024 Mar 25;15:1373341. doi: 10.3389/fneur.2024.1373341 (PMC10999640; doi:10.3389/fneur.2024.1373341)
Supplement: Supplementary file 1 [file Table_1.DOCX]

**Supplementary Table 1. Patient diagnoses.**

| **Diagnosis** | **Number of participants** |
| --- | --- |
| Alzheimer's Disease;  Familial Alzheimer’s Disease | 34 |
| Cognitively Impaired, Not Demented/Mild Cognitive Impairment | 6 |
| Vascular Dementia | 2 |
| Vascular-Cognitively Impaired, Not Demented/Vascular Cognitive Impairment/ Stroke/Transient Ischemic Attack | 6 |
| Alzheimer's Disease with Cerebrovascular/ Small Vessel Disease | 12 |
| Frontotemporarl Dementia, behavioral variant, incl.FTD+Motor Neurone Disease | 6 |
| FTD language variant (x3 types): i. FTD non-fluent progressive aphasia ii. FTD fluent /semantic dementia iii. FTD Logopenic variant/ logopenic progressive aphasia | 11 |
| Lewy Body Dementia | 1 |
| Cortical Basal Syndrome/ Degeneration | 5 |
| Not Yet Diagnosed | 1 |
| Uncertain Diagnosis (e.g. A vs. B) | 9 |
| Alzheimer's Disease and Lewy Body Disease/ Dementia with Lewy Bodies | 4 |
| Mixed disease (confirmed by autopsy) | 1 |
| Atypical Alzheimer's Disease (e.g. AD language variant, AD frontal variant) | 8 |
| Alzheimer's Disease and Vascular Dementia | 3 |
